# Application of Deep Learning Methods for Binarization of the Choroid in Optical Coherence Tomography Images

**DOI:** 10.1167/tvst.11.2.23

**Published:** 2022-02-14

**Authors:** Joshua Muller, David Alonso-Caneiro, Scott A. Read, Stephen J. Vincent, Michael J. Collins

**Affiliations:** 1 Queensland University of Technology (QUT), Contact Lens and Visual Optics Laboratory, Centre for Vision and Eye Research, School of Optometry and Vision Science, Kelvin Grove, Queensland, Australia

**Keywords:** image analysis, choroidal vascularity index, stromal, luminal, classification

## Abstract

**Purpose:**

The purpose of this study was to develop a deep learning model for automatic binarization of the choroidal tissue, separating choroidal blood vessels from nonvascular stromal tissue, in optical coherence tomography (OCT) images from healthy young subjects.

**Methods:**

OCT images from an observational longitudinal study of 100 children were used for training, validation, and testing of 5 fully semantic networks, which provided a binarized output of the choroid. These outputs were compared with ground truth images, generated from a local binarization technique after manually optimizing the analysis window size for each individual image. The performance was evaluated using accuracy and repeatability metrics. The methods were also compared with a fixed window size local binarization technique, which has been commonly used previously.

**Results:**

The tested deep learning methods provided a good performance in terms of accuracy and repeatability. With the U-Net and SegNet networks showing >96% accuracy. All methods displayed a high level of repeatability relative to the ground truth. For analysis of the choroidal vascularity index (a commonly used metric derived from the binarized image), SegNet showed the closest agreement with the ground truth and high repeatability. The fixed window size showed a reduced accuracy compared to other methods.

**Conclusions:**

Fully semantic networks such as U-Net and SegNet displayed excellent performance for the binarization task. These methods provide a useful approach for clinical and research applications of deep learning tools for the binarization of the choroid in OCT images.

**Translational Relevance:**

Deep learning models provide a novel, robust solution to automatically binarize the choroidal tissue in OCT images.

## Introduction

Binarization of optical coherence tomography (OCT) images of the choroid can provide valuable information regarding the anatomical structure of this tissue by discriminating vascular luminal areas (low/hypo reflectivity regions) from interstitial areas (high/hyper reflectivity regions), that correspond to the vascular wall and choroidal stromal connective tissue. Previous studies have demonstrated that changes in the choroidal blood vessels and nonvascular stromal tissue are involved in the pathogenesis of several ocular conditions, such as age-related macular degeneration, polypoidal choroidal vasculopathy, central serous chorioretinopathy, diabetes, and myopic macular degeneration.^1^^–^^6^ Therefore, data from binarization analysis can be used to quantify the choroidal structure, providing a vascularity index or ratio of luminal to stromal areas in the tissue, which allows for a more comprehensive characterization of the choroid's anatomical and physiological properties than is provided by traditional thickness information.^7^

Although optical coherence angiography is emerging as a new modality to capture ocular angiography information, this imaging modality has primarily been used for mapping retinal vasculature, because current devices are unable to comprehensively map the complete choroidal dynamics across the full thickness of the tissue.^8^ Thus, to extract the complete choroidal tissue information, binarization techniques need to be applied to high resolution static OCT images. Binarization techniques convert the grey-scale range of intensity values in the OCT image to two values (i.e. black and white), and this is typically achieved by selecting a threshold which determines the classification boundary. Traditional binarization methods can be classified as either global or local approaches.^9^ The global approach works on the entire image and obtains a single threshold value for the whole image. Although this method is commonly used, it tends to be less effective on images with varying levels of noise, complex structure, poor quality, and multiple contrast levels (i.e. different contrast across the image),^10^ which are features commonly encountered in OCT images of the posterior segment of the eye. To solve these problems, local binarization methods can be used. These methods subdivide the image into smaller regions (also known as windows) and determine the threshold value of each of these regions in the image independently, which overcomes many of the limitations of global methods. Both of these “traditional binarization methods” have been widely applied to OCT binarization.^11^

For completeness and to provide context to the proposed analysis, some recent studies in this field are summarized here. The binarization of OCT B-scan images to characterize the choroidal vascular features in different cohorts has been performed in several studies, with a variety of different methods used, including a range of global and local thresholding techniques. Initial work proposed the use of a single constant threshold value^12^ or the global Otsu method to analyze the vasculature features.^13^ A study by Ruiz-Medrano^14^ used more sophisticated image preprocessing techniques (image denoising and exponential and nonlinear enhancement to increase the image contrast to better visualize various structures) followed by thresholding using the global Otsu's algorithm. However, many of the studies have used some form of local binarization method, particularly the local Niblack binarization method, which has been commonly applied to study the proportion of luminal and stromal areas of the normal choroid in OCT images.^15^^,^^16^ This Niblack local thresholding method has been used to investigate changes in vascular characteristics associated with different pathologies.^17^^,^^18^ It is worth noting that despite the potentially important influence of window size upon binarization outcomes, the window size is normally not reported in these studies using local binarization methods. Another study^19^ segmented the choroidal vessels using an enface OCT image, which were labeled by adaptive thresholding and each subsequent frame was connected via segment propagation from the frame above, and used as the reference for the next frame.

In recent years, deep learning methods have been applied to a range of different OCT image analyses. Deep learning algorithms are particularly suitable for image segmentation and classification tasks and have demonstrated state of the art performance for a range of applications involving analysis of the choroid in OCT B-scans. However, most of these methods have focused on the segmentation of the choroid to derive thickness measures^20^^–^^22^ with the application of deep learning strategies for choroid binarization receiving much less attention. Using swept source OCT images, Zhang and colleagues^23^ proposed the use of a deep learning residual U-Net to identify the choroidal boundaries, however, the authors applied Niblack's auto-local threshold algorithm to extract the vasculature information. Liu et al.^24^ proposed the use of RefineNet, a fully convolutional network, to label the vasculature within a B-Scan. RefineNet showed a greater agreement with two independent observers than the interobserver agreement and the method was deemed suitable to segment the choroidal vessels from swept source OCT images. Some other examples of binarization include multiscale 3D edge filtering and projection,^25^ en-face segmentation,^26^ and adaptive thresholding^27^ methods.

Given the potential of deep learning methods for medical image analysis and the rich amount of information encapsulated in the choroidal region of OCT images, this study explores the application of a range of deep learning techniques for the binarization of spectral domain OCT images of the choroid. A large dataset of images is used for training, following careful binarization to optimize the window size of the local method, which has been unexplored in previous studies.

## Methods

### Subjects and Procedures

The OCT images used in this study were collected as part of a longitudinal clinical study which examined changes in choroidal thickness and axial length in a population of children with a range of refractive errors. The protocol has been described in detail elsewhere,^28^^,^^29^ and, here, only a brief summary of the data collection procedure is provided. The study was an 18-month prospective, observational longitudinal study which included 100 children aged between 10 and 15 years. All children enrolled in the study exhibited best corrected visual acuity of logMAR 0.00 or better in each eye, and no history or evidence of significant ocular disease. Forty children were classified as myopic (mean subjective spherical equivalent refraction [SER] −2.39 ± 1.51 D) and 60 as non-myopic (mean subjective SER +0.35 ± 0.31 D). The myopic and non-myopic children were well matched for both age and sex. After baseline measurements, subsequent measurement sessions were conducted every 6 months over an 18-month period. Approval from the Queensland University of Technology human research ethics committee was obtained prior to commencement of the study, and all parents provided written informed consent for their child to participate, and all children provided written assent. All participants were treated in accordance with the tenets of the Declaration of Helsinki.

OCT images were collected at each session with the Heidelberg Spectralis SD-OCT instrument (Heidelberg Engineering, Heidelberg, Germany). This device uses an 870-nm super luminescent diode for OCT imaging with a scanning speed of 40,000 A-scans per second, to provide chorioretinal OCT images with an axial resolution of 3.9 μm and transverse resolution of 14 μm. At each study visit, children had 2 series of OCT images of their right eye collected using a high-resolution six line “star” scanning protocol, consisting of six 30 degrees long (approximately 8.5 mm), radial line scans centered on the fovea, each separated radially by 30 degrees. All OCT images were captured using the instrument's enhanced depth imaging (EDI) mode to optimize choroidal visibility, as well as automatic real-time eye tracking to average 30 B-scans and reduce noise. Scans were collected using the instrument's “Auto Rescan” feature, to register follow-up OCT scans to the same retinal location as the baseline measurements.

### Data Set Preparation

For this study, a subset of the entire dataset was used for the analysis, consisting of data from 100 participants over the 4 visits. Only the vertical (superior to inferior) and horizontal (nasal to temporal) B-scans obtained at each visit with repeated measures were used, because the architecture and topographical distribution of the vessels and variations in choroidal thickness between these two meridians provided a diversity of vascular features between scans. In total, the data set contained 1632 OCT B-scan images with their corresponding choroidal boundary delineation and binarized images for the choroid with the 2 categories of interest (lumen and interstitial). The choroidal tissue was demarcated by the inner choroidal boundary (ICB) and the outer choroidal boundary (OCB), where the ICB coincides with the outer boundary of the retinal pigment epithelium/Bruch's membrane complex and the OCB marks the transition between the choroid and sclera. These boundaries were previously segmented using an automated method and manually corrected by an expert observer if needed, as described previously.^28^^,^^29^

**Figure 1.**
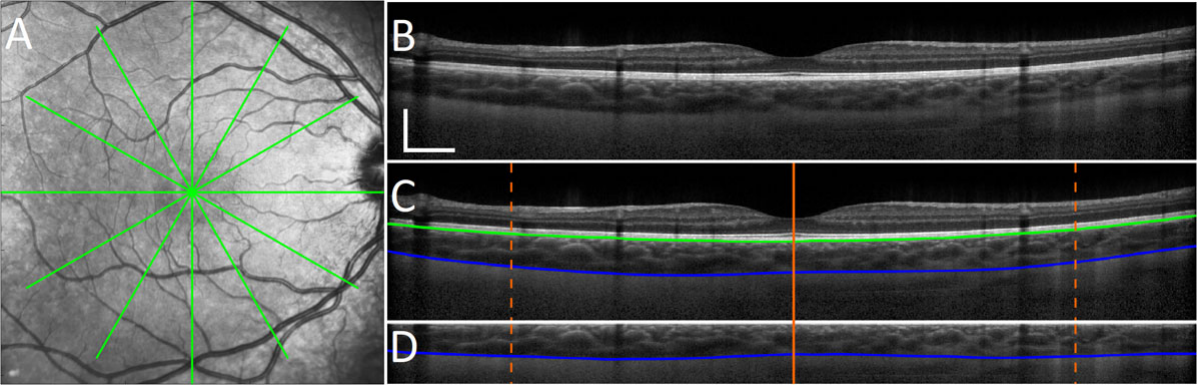
Example of an en face image (**A**) and a single B-scan (**B**) captured with the OCT using the instrument's high resolution scanning protocol with the two segmented (**C**) boundaries of interest, the inner choroidal boundary (ICB; *green line*) and outer choroidal boundary (OCB; *blue line*). The flattened, cropped version of the image encapsulating the choroidal region used for analysis is also shown (**D**), with the sclera located below the blue line. The scales bars (B subplot) represent approximately 0.5 mm (horizontal and vertical).

Because binarization methods typically work on a rectangular region of the image, and the posterior segment of the eye is naturally curved, the choroidal region of interest, which will be later used to discriminate the vascular-interstitial threshold, needs to be flattened to ensure the analysis encompasses the complete section of the choroid. To achieve this, the image shape was transformed (flattened), using a similar approach to image flattening used in OCT layer segmentation methods.^30^^,^^31^ The ICB, which corresponds to the retinal pigment epithelium layer, was used as a reference to compensate for this curvature and flatten the image, by shifting each A-scan to move the ICB boundary to the top part of the image, leaving only the choroidal section on the top of the B-scan. After flattening the image, a rectangular region which contains the choroid was cropped. The height of the region to be cropped was set to be the maximum scleral height within the image data set, which was then applied identically to each image. This cropped height was calculated to be 59 pixels of the original height of 200. Figure 1 shows an example of the B-scan flattening; where the original B-scan Figure 1B was first segmented to obtain the boundaries of interest (see Fig. 1C) and then flattened using the ICB estimate and cropped (see Fig. 1D). Prior to binarization, the 6 mm subfoveal region of the choroid was extracted from the B-scan (delineated in the figure with the orange solid lines).

### Image Binarization With Local Methods

**Figure 2.**
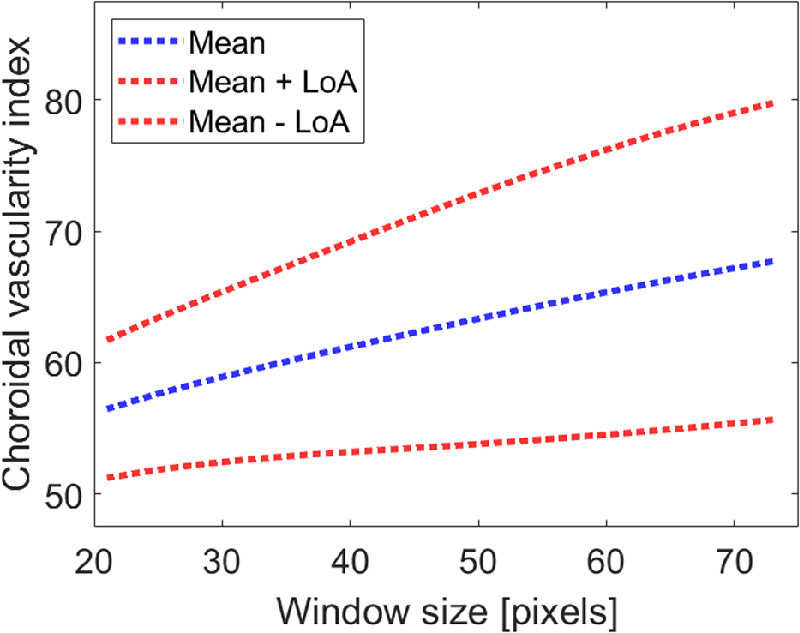
Choroidal vascularity index (CVI) as a function of window size in the NiBlack local binarization method. Blue and red dotted lines represent the mean and 95% limits of agreement (LOA), respectively.

Numerous methods have been proposed for choroidal image binarization, particularly local binarization techniques. These techniques typically compute a threshold value for each pixel by shifting a rectangular window across the image. The threshold T(i,j) for the center pixel (i,j) of the window is computed using a mathematical formula specific to the particular method. For these methods, the user must select the size of the rectangular analysis window; in the case of OCT image analysis for assessing choroidal vascularity, the size of the analysis region (window) should be large enough to encompass both vascular and interstitial areas but small enough to preserve local tissue details. Thus, the selection of an appropriate window size for analysis is critical, however, the impact of this factor upon OCT choroidal binarization analyses has not been addressed previously, with most of the previous studies using a fixed window size. To illustrate this effect, Figure 2 shows the change in the choroidal vascularity index (CVI; a commonly used metric that quantifies the ratio of the luminal area to the total choroidal area in a binarized OCT image) as a function of the selected window size for each participant. This analysis was conducted using data from a single visit, and a range of window sizes from 20 to 75 pixels. The mean ± standard deviation increase in the CVI associated with a change in window size from 20 to 75 pixels was 11.23 ± 4.41% (range = 1.95 to 20.51%).

Niblack's binarization method^32^ has been applied to OCT choroidal imaging in a number of studies.^1^^,^^17^ The method uses the following formula to calculate the threshold:

$$\begin{array}{c} T\left(i,j\right)=m\left(i,j\right)+k\sigma \left(i,j\right) \end{array}$$

where m and σ are the mean and standard deviation value of the pixels inside the window center at (i,j)^33^ and k acts as a “weight” of the standard deviation to adjust how much of the total object boundary is taken as a part of the object. For example, in text binarization, small (negative) k values will produce thick and unclear strokes, whereas large (positive) k values will result in slim and broken strokes. The quality of binarization depends on the parameter k and the size of the sliding rectangular analysis window. The main limitation of this method is that it produces a large amount of noise in the empty windows (i.e. when only a single class such as the lumen or stroma is present). To minimize complexity and ensure the selection of k does not bias the results, the parameter k was empirically set to −0.05 for the remaining analyses.

Given the effect that window size has on the binarization of the image, and the need for a training data set to develop an automatic deep learning method for binarization of the choroid, a graphic user interface was developed to allow an observer to manually select the optimal window size to visualize the choroidal vascular structure. The interface displayed a 6 mm standard reflectivity image and its equivalent binary. Initially the windows size was set to a minimum (5 × 5 pixels) and an experienced observer was asked to adjust the window size using a slide bar to determine the optimum window size that suppressed binarization noise while preserving local tissue details. This process was undertaken on the standard reflectivity images using the same pediatric dataset of B-scans from two scanning orientations (horizontal and vertical) from all four visits. The final, optimized binarized choroidal images from this analysis were then used to train the deep learning methods under investigation. The window size selection procedure was repeated for the scans from the first visit, and the repeatability of this procedure was determined using an intraclass correlation and Bland-Altman analysis.

### Deep Learning Network

**Figure 3.**
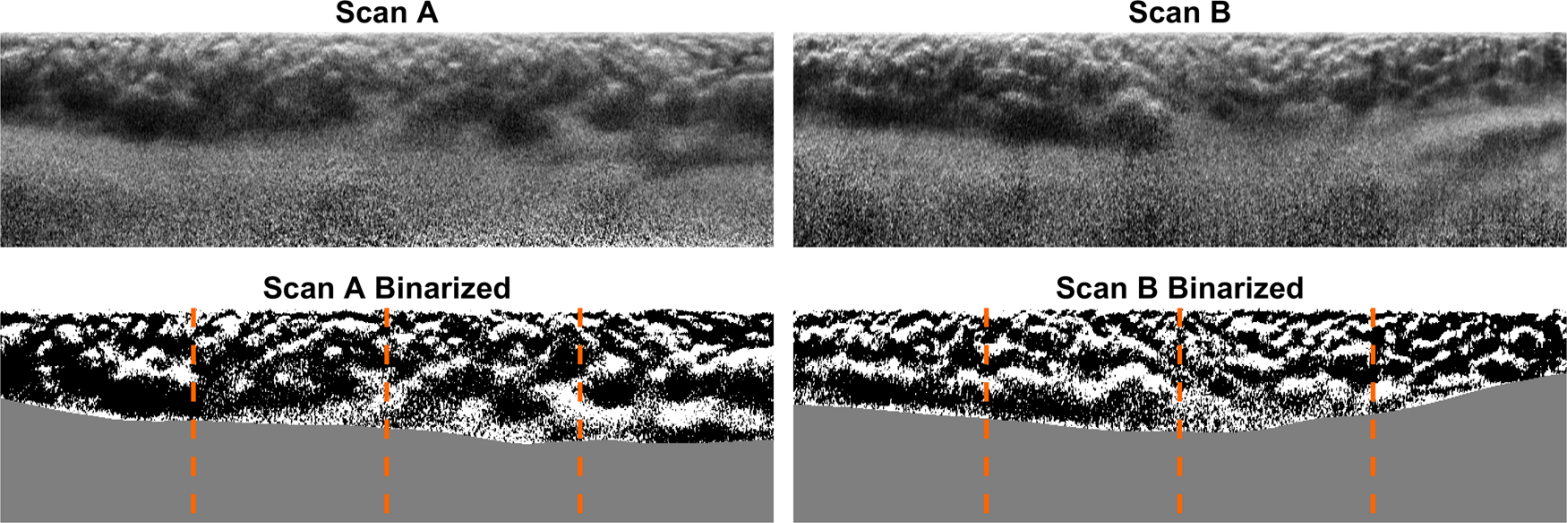
Example of a horizontal (**A**) and vertical (**B**) OCT scans (standard reflectivity), and their corresponding binarization image in which the scleral region has been masked (*grey*). The *orange dotted lines* delineate the four nonoverlapping regions used to satisfy the input parameters of the network.

The binarized images, which were obtained after the optimal window size was selected for each individual B-scan, together with the scleral mask image (determined from the segmentation of the OCB) were combined to create the ground truth images for the entire data set (Fig. 3). This was done such that each ground truth image conveyed three unique categories (lumen, interstitial, and scleral). Because the ground truth binarized image is a 3-channel image, the first 2 choroidal-tissue categories are denoted as the greyscale values of 0 for the interstitial and 255 for the lumen, respectively. The lower scleral region, which encompasses the scleral tissue adjacent to the choroid and is not of significance to this study, was treated separately. Given that the image contains a portion of the sclera, and to allow the network to train accurately on the remaining categories, the scleral region was masked in the ground truth image as a color value (R-127, G-0, and B-0). This masking technique creates a “highlighted region” for the network, allowing the networks to train near-perfectly in this region category. If the choroidal boundaries are not present, particularly the OCB that is more challenging to be segmented, then the scleral region could be assigned to a category and classified by the network. The effect on performance of not “masking the scleral region” was also examined.

The networks considered in this work had minimum input size requirements. To standardize the data, both the input OCT scan images and the ground truth images were cropped into four nonoverlapping sections to satisfy the input parameters of each network being trained. This resulted in each original (224 × 896 pixels) image being split into four (224 × 224 pixels) images, and the same input size was used across all networks. The input to the network is a three channel input (224 × 224 × 3 pixels), which is also used to mask the sclera.

The images were distributed amongst three data sets consisting of 60% training, 20% validation, and 20% testing. Although the subject allocation among the data sets was randomly allocated, the specific order of the participants from the original data set was preserved within these subsets, such that one individual participant's scans over the four visits will only appear within one of these subsets. The proportions of categories for the ground truth images within each dataset are denoted in Table 1. One-way ANOVA revealed a statistically significantly smaller percentage of the scleral region within the training data set compared to the testing and validation datasets (*P* < 0.001). This reflects a slightly greater choroidal thickness in the training dataset, which resulted in a significantly greater percentage of interstitial and lumen regions in the training data set also (all *P* < 0.001). However, there was no significant difference in the CVI between the data sets (*P* = 0.59), indicating similar choroidal vascular characteristics across the three datasets.

**Table 1.** Dataset Summary, Including the Number of Subsection Images (224 × 224 Pixels) Per Set and the Mean ± Standard Deviation of the Three Categories Within the Image

		Category (Mean ± Standard Deviation, %)			
	Number of Image Subsections	Interstitial	Lumen	Scleral	CVI
Training	3488	38.86 ± 8.43	25.28 ± 5.36	35.84 ± 12.95	60.51 ± 3.51
Validation	560	32.63 ± 8.44	20.91 ± 5.60	46.46 ± 13.64	60.92 ± 3.08
Testing	560	33.78 ± 7.10	22.60 ± 6.65	43.62 ± 13.24	60.25 ± 3.35

### Networks and Training Regime

To better understand the potential of deep learning sematic segmentation methods for the binarization of the choroid, a range of networks were evaluated. Specifically, three different well-established network architectures were used: DeeplabV3+, U-Net, and SegNet. All networks were trained to classify three classes (interstitial, lumen, and scleral).

DeepLabV3+ is a semantic segmentation network developed by Google, which incorporates atrous convolutions and atrous spatial pyramid pooling (ASPP) to increase output feature maps and decrease the total computational cost of the network.^34^ Atrous convolution behaves similarly to standard convolution except with specified gaps between each pixel of the convolution window, allowing a larger feature of the image to be included in each window. ASPP captures feature maps at multiple scales by resampling feature layers at multiple rates, thereby capturing feature map context at different scales. DeepLabV3+ has the capability to incorporate pre-weighted networks. In this work, three pre-weighted networks were tested: ResNet18, ResNet50, and MobileNetV2. This network has been applied to OCT image analysis for retinal disease segmentation.^35^

The second network is U-Net, a popular semantic segmentation network that has a distinctive “U shaped” encoder-decoder architecture with skip connections between the contracting and expanding path. U-Net has been widely applied to OCT image analysis of the posterior eye, including choroidal^20^ and retinal segmentation.^36^ The number of filters is doubled at each subsequent pooling layer to allow a greater level of feature encoding. For this work, an encoder depth of four was used.

The third network, SegNet, is also a semantic segmentation network that incorporates an encoder-decoder architecture followed by pixel-wise classification.^37^ Encoding is achieved through 13 convolutional layers followed by 5 max pooling layers, in which the boundary information is stored as max-pooling indices before images are subsampled. This boundary information is utilized in the decoder network, in which the memorized max pooling indices are used to accurately up sample. For this work, an encoder depth of four was used for the SegNet networks. This network architecture has also been applied to choroidal^38^ and fluid segmentation^39^ in ocular OCT images.

To standardize the result and focus on the effect of the network, the training parameters (optimizer, learning rate, number of epochs, and batch-size) were kept constant across the different networks. Each network was trained three times with an identical set of hyper-parameters, specifically an ADAM optimizer was used, with a cross-entropy loss function. The learning rate was set to a constant value of 10^−4^. Each model was trained for a total of 15 epochs, with data shuffle for every epoch and a mini-batch size of 10. It is worth noting that the number of predefined parameters of the network differs between architectures. The SegNet and DeepLabV3+ network models were pre-initialized with layers and weights from a pretrained model, which were trained to the specific OCT binarization task.

To further validate the method, against the binarization technique that has typically been used in previous studies, the data set was also binarized using the NiBlack technique with a fixed window size. Although, as previously mentioned, this may not be the optimal binarization method for OCT images, it facilitates the comparison of this standard technique with the proposed deep learning methods. For this analysis, a fixed window size of 51 pixels was selected, because it represents the mean value of the range of window sizes that were manually selected.

### Evaluation

The evaluation of the methods was assessed by extracting a range of metrics, which can be divided into two main categories: the metrics associated with the accuracy of the binarization (comparison to the ground truth) and the metrics associated with the repeatability (of the binarization within the two repeated images that were captured for each participant at each visit in the study). It is worth noting that the performance evaluation is conducted using the original B-scan resolution, so the results are not biased by any of the preprocessing steps.

For accuracy, the evaluation of performance involved calculating two metrics: the total and per-class accuracy, and the intersection over union (IOU). The accuracy of the testing data set was calculated using the following formula:

$$\begin{array}{c} Accuracy=\;\frac{T_{P}}{\left(T_{P}+F_{N}\right)} \end{array}$$

where T_P_ is the number of true positives and F_N_ is the number of false negatives, calculated in a per-class basis or globally depending on the accuracy reported. The IOU is the ratio of correctly classified pixels to the total number of ground truth and predicted pixels of that class, given by the following formula:

$$\begin{array}{c} IoU=\;\frac{T_{P}}{\left(T_{P}+F_{P}+F_{N}\right)} \end{array}$$

where F_P_ is the number of false positives. This metric is calculated on a per-class basis as the ratio of true positives to the sum of ground truth and predicted pixels within a particular class, per image as the average class score for an individual image, and on the entire image dataset as an average of each IOU score for each class. A repeated measures ANOVA with Bonferroni adjusted post hoc pairwise tests was conducted to compare the accuracy and IOU across the methods.

Taking into consideration that all the scans included in the data set have “follow-up” scans taken at the same visit aligned at the same retinal location, a repeatability analysis was also performed comparing the two scans collected at the same visit on each participant. The repeatability was calculated as the absolute difference in the proportion of pixels for each category, given by the following formula:

$$\begin{array}{c} R=\;\frac{\left|P_{1}-\;P_{2}\right|}{p}\times \;100 \end{array}$$

where *P*_1_ and *P*_2_ are the number of correctly classified pixels for scans 1 and 2 respectively for a given category, *p* is the number of total pixels in an image (224 × 896 pixels), and *R* provides a repeatability metric given as a percentage difference between scans 1 and 2 for each category (with a percentage of 0 indicating perfect agreement between 2 repeated scans). This metric was used to gage the repeatability performance of the networks in comparison to the ground truth which are the manually binarized images. The specific class proportion (%) metrics between scan 1 and 2 were used to assess the correlation, and Bland-Altman plots were generated to assess the method repeatability versus the ground truth (based on local binarization).

A quantitative index, called the CVI, has been proposed to examine the vascular status of the choroid.^40^ The CVI measures the ratio of blood vessels in the luminal area to the total choroidal area (i.e. the sum of the total number of white pixels over the total number of pixels in the choroidal area), with a greater CVI indicating a higher percentage of blood vessels per area of the choroid. The mean CVI for scans 1 and 2, their difference and 95% limits of agreement (LOA) were also calculated to assess the repeatability of this commonly reported metric.

## Results

### Repeatability Analysis Ground Truth Dataset

**Figure 4.**
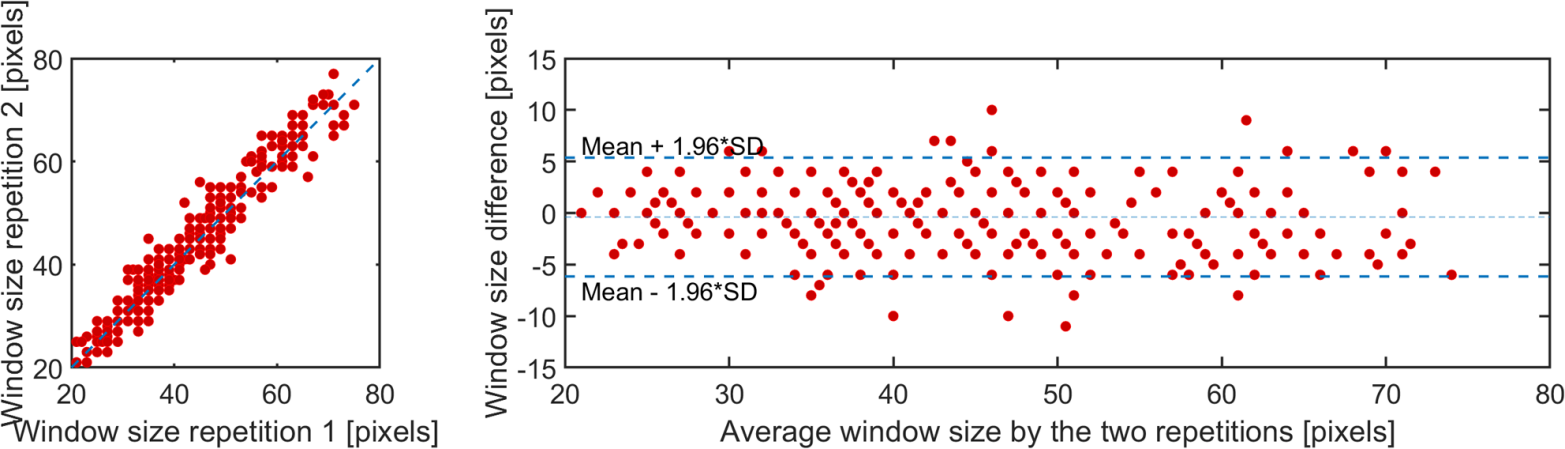
Correlation between the repetitions for the manual selection of window size (*blue line* indicates the line of equality between the two repeats) (*left panel*). Bland–Altman plot of the difference versus the mean of the two repetitions (*right panel*).

To provide an estimate of the repeatability of the manual window selection for the choroid local binarization method, the observer repeated the selection of the window size on the scans from visit 1 twice, and the results were compared using an intraclass correlation (ICC) and Bland-Altman analysis. The 2 repetitions were highly correlated with an ICC = 0.968 (Fig. 4, left panel), and a paired *t*-test comparing the values obtained for the 2 window size repetitions revealed no statistically significant difference (*P* = 0.66). Thus, the intra-observer repeatability for the manual selection of the window size showed an excellent agreement. For the Bland-Altman analysis, the mean difference between the two repetitions was −0.40 ± 2.96 pixels (95% LOA −6.33 to 5.53 pixels; see Fig. 4, right panel), and the mean absolute difference was 2.25 ± 1.96 pixels (95% LOA −1.68 to 6.19 pixels). No statistically significant proportional difference associated with the mean window size was observed (r^2^ = 0.03, *P* = 0.09).

### Network Performance and Repeatability

Accuracy metrics were collated for each network over the three independent runs as well as for the fixed window binarization method. A summary of the different accuracy metrics can be found in Table 2. All the tested networks provided reasonable binarization performance compared to the ground truth, with a total accuracy above 92% for all the five tested networks. However, it is clear across all the different metrics that the three variations of DeepLabV3+ (ResNet18, ResNet50, and MobileNetV2) did not perform as well as the U-Net and SegNet. Overall, U-Net and SegNet displayed very comparable performance across the metrics, however, the U-Net provided slightly superior per-class accuracy with values of 97.06% and 93.57% for lumen and interstitial categories, respectively. Overall, these metrics demonstrate the potential of deep learning methods for the binarization of the choroid in OCT images. While assessing the performance of the fixed window size, the overall accuracy metrics show a clear negative impact on performance compared to the U-net and SegNet deep learning methods. Poorer performance is particularly evident for the detection of the interstitial region that presents a low per-class accuracy. Supplementary Figure S1 provides a performance comparison of the fixed window size ranging between 20 and 70 pixels and compares the mean accuracy versus the values of the U-Net. A difference in both the mean accuracy as well as the spread of the values between the fixed window size approach and U-Net can be observed.

**Table 2.** Summary of the Network Performance in Terms of Accuracy

			Per-Class Accuracy, %		
	Total Accuracy, %	IoU, %	Lumen	Interstitial	Sclera
U-Net	96.88 ± 1.00	94.28 ± 1.62	97.06 ± 0.90	93.57 ± 2.77	99.99 ± 0.00
DeepLabV3- ResNet18	92.39 ± 1.04	86.69 ± 1.56	92.25 ± 1.47	84.94 ± 2.84	99.97 ± 0.02
DeepLabV3- ResNet50	92.49 ± 1.14	86.79 ± 1.63	92.06 ± 1.49	85.44 ± 3.47	99.97 ± 0.02
DeepLabV3- MobileNetV2	92.65 ± 1.03	86.84 ± 1.51	91.26 ± 1.47	86.70 ± 2.97	99.97 ± 0.02
SegNet	96.13 ± 0.90	92.97 ± 1.41	96.46 ± 0.81	91.93 ± 2.61	99.99 ± 0.00
Fixed Window 51	94.05 ± 1.77	90.09 ± 4.23	96.68 ± 1.66	85.47 ± 8.16	100.00 ± 0.00

Values are the mean ± standard deviation, which is calculated as the deviation of the mean for the three independent tested networks. The scleral region was masked to facilitate detection.

Repeated measures ANOVA revealed a statistically significant effect of the analysis method upon total accuracy and IOU (both *P* < 0.001). Pairwise comparisons showed that U-Net exhibited significantly greater total accuracy and IOU compared to all other tested methods (all *P* < 0.001). The fixed window size exhibited statistically significantly worse performance (both accuracy and IOU) compared to both the U-net and SegNet methods (both *P* < 0.001). To understand the impact on the manual correction of the boundaries on the performance, the uncorrected and corrected data sets were compared, both binarized using the optimal window size. All metrics showed that the correction of the boundaries had minimal to no effect on the metrics, with an accuracy of 99.7%, and IOU of 99.5% and interclass accuracy over 99.5%.

A more subtle difference between the methods was observed for the repeatability metrics compared to the accuracy outcomes (Table 3). All of the metrics provided values that closely match the repeatability of the ground truth. Despite significantly better performance in accuracy, U-Net's repeatability values for the interstitial and lumen are higher (less repeatable) than the other methods, although the percentage differences are small. Interestingly, the networks that demonstrated the best repeatability on average (DeepLabV3+ variations) had inferior accuracy. Unlike the accuracy metrics, the repeatability performance for the fixed window size is comparable to the other methods.

**Table 3.** Repeatability Metric Per Class for all Five Tested Networks Versus the Ground Truth (Based on Local Binarization)

	Interstitial, %	Lumen, %	Sclera, %
U-Net	0.82 ± 0.99	0.66 ± 0.74	0.82 ± 0.66
DeepLabV3- ResNet18	0.78 ± 0.95	0.62 ± 0.66	0.82 ± 0.82
DeepLabV3- ResNet50	0.79 ± 0.88	0.67 ± 0.61	0.89 ± 0.66
DeepLabV3- MobileNetV2	0.76 ± 0.83	0.69 ± 0.62	0.89 ± 0.66
SegNet	0.77 ± 0.87	0.63 ± 0.64	0.82 ± 0.66
Ground Truth	0.73 ± 0.82	0.62 ± 0.54	0.82 ± 0.66
Fixed Window 51	0.70 ± 0.82	0.62 ± 0.56	0.82 ± 0.66

Values are presented as the mean ± standard deviation, which is calculated as the percentage deviation of the mean for the three independent tested networks. The scleral region was masked to facilitate detection.

It is also worth noting the metrics related to the sclera (see Tables 2, 3), which demonstrates that for all networks the accuracy and repeatability for the scleral detection is excellent. This confirms that the masking strategy works well to support the detection of this region. The small variation in the scleral region (see Table 3) is mostly related to the boundary placement between consecutive scans. If the scleral region is not masked in the OCT image, the network could be tasked to classify all three regions. The accuracy and repeatability results when the scleral region is not masked, are provided in Supplementary Tables S1 and S2. The findings in terms of network performance are similar to the masked version, with the U-Net and SegNet achieving a superior performance. However, overall, the results are worse than the masked version, which is to be expected because the features in the scleral region are similar to those in the choroid in some B-scans. All networks exhibited high accuracy in classifying the scleral region. However, classification of the scleral region presents a trade off in accuracy among the interstitial and luminal regions. For example, for U-Net, the unmasked luminal and interstitial accuracies were 94.07 ± 5.38 and 84.42 ± 5.91, respectively, compared to masked luminal and interstitial accuracies of 97.06 ± 0.90 and 93.57 ± 2.77, respectively. Therefore, where possible, the masked strategy may be the more viable and accurate method to consider.

### CVI Performance and Repeatability

**Figure 5.**
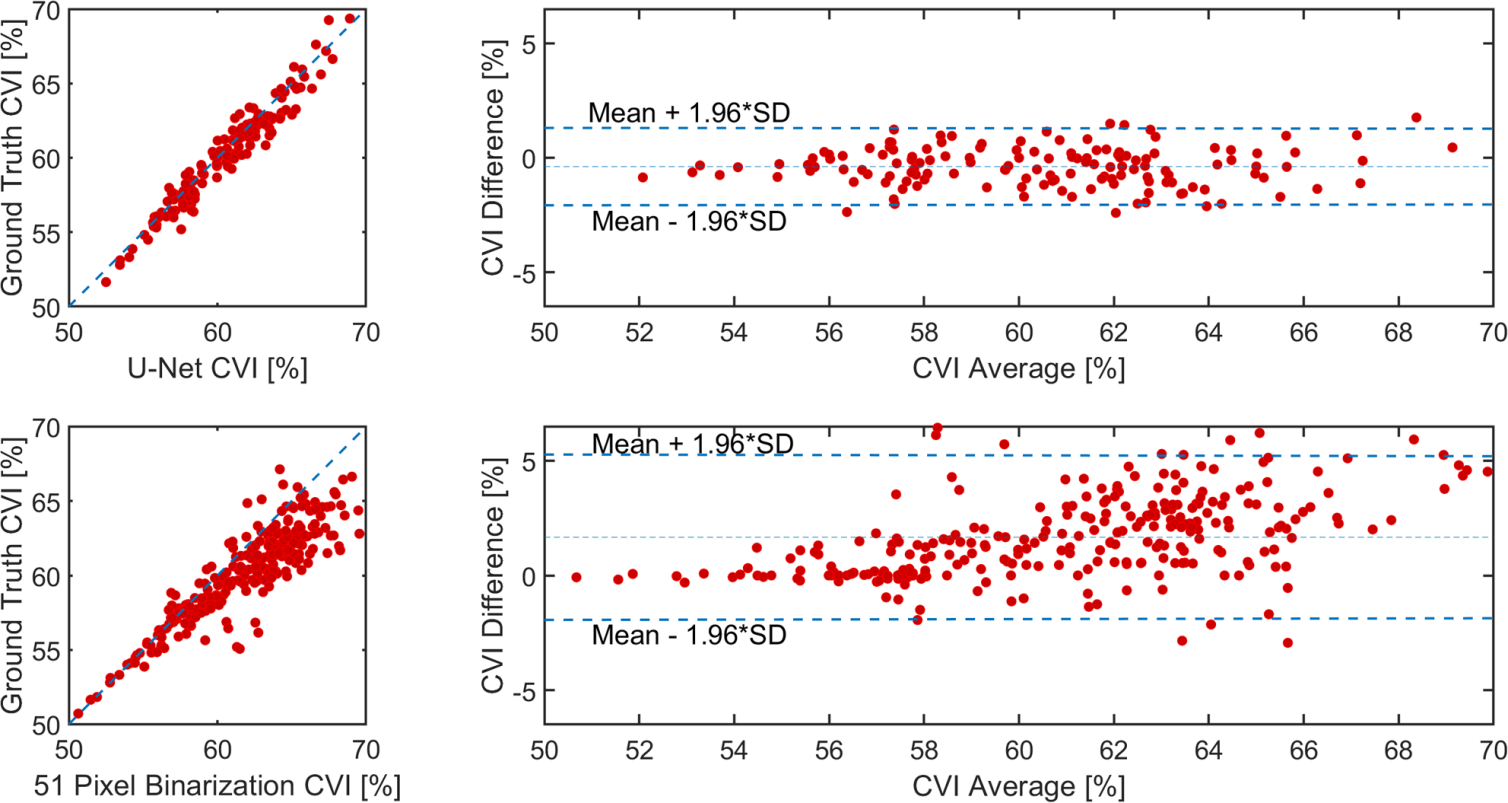
Comparison of the choroidal vascularity index (CVI, %) between U-Net and Ground Truth (*top*), and fixed window binarization 51 and Ground Truth (*bottom*).

**Figure 6.**
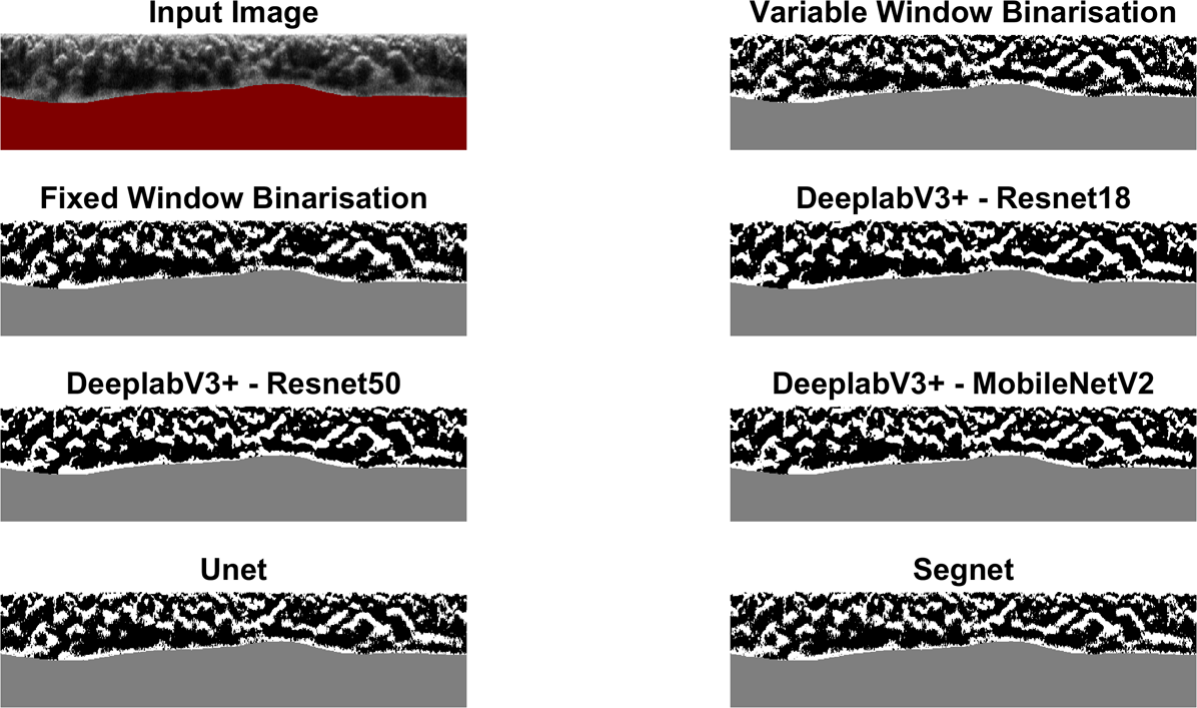
Input image with the masked scleral region, with its corresponding ground truth (Variable Window) and fixed window size (51 pixels), as well as the output binarization examples for all the tested networks.

Tables 4 and 5 summarize the accuracy and repeatability of the commonly used CVI metric derived from each of the methods. All networks showed minor differences compared to the ground truth values, with the largest differences (>3%) observed for the fixed window size binarization. Repeatability was very similar across the different methods. Overall, the results demonstrate the potential of the deep learning methods for this particular image analysis application. Given the similar level of repeatability between the methods, the methods with the highest accuracy (U-Net and SegNet) may be the preferred option for future applications of this technique. Similar to the overall accuracy metrics, the CVI does not compare well when the fixed window size method is applied. Figure 5 shows the correlation (ground truth) versus the U-net (top) and fixed window size (bottom), and there is a clear underestimation of the CVI compared to the ground truth with increasing CVI and a widening of the limits of agreement for this commonly used fixed window size method. The close agreement between methods can be observed in the example provided in Figure 6, which shows the different binarization outputs for the same B-scan.

**Table 4.** Accuracy Metrics for the Choroidal Vascular Index

	CVI-S1, %	CVI-S2, %	Mean Absolute Difference [S1/2–GT1/2], %
U-Net	60.99 ± 3.74	61.10 ± 3.69	1.05 ± 0.80
DeepLabV3- ResNet18	61.09 ± 3.71	61.23 ± 3.62	1.05 ± 0.75
DeepLabV3- ResNet50	61.18 ± 3.57	61.30 ± 3.46	1.09 ± 0.79
DeepLabV3- MobileNetV2	60.49 ± 4.10	60.56 ± 3.94	0.96 ± 1.04
SegNet	60.59 ± 3.38	60.78 ± 3.26	0.75 ± 0.55
Ground Truth	60.21 ± 3.39	60.28 ± 3.32	N/A
Fixed Window 51	63.64 ± 5.28	63.38 ± 5.18	3.47 ± 2.63

S1 and S2 represent the two scan repetitions, whereas GT denotes the ground truth.

N/A, not applicable.

**Table 5.** Repeatability Metrics for Choroidal Vascularity Index (CVI), Including the Intraclass Correlation Coefficient (ICC), the Mean Absolute Difference (Scan 1–Scan 2), Mean Difference, and 95% Limits of Agreement (LoA)

	ICC	Mean Absolute Difference, %	Mean Difference ± 95% LoA, %
U-Net	0.928	0.98 ± 1.01	0.11 ± 2.76
DeepLabV3- ResNet18	0.932	0.98 ± 0.92	0.13 ± 2.63
DeepLabV3- ResNet50	0.931	0.98 ± 0.86	0.12 ± 2.56
DeepLabV3- MobileNetV2	0.944	1.03 ± 0.86	0.07 ± 2.63
SegNet	0.926	0.93 ± 0.87	0.18 ± 2.49
Ground Truth	0.935	0.89 ± 0.82	0.07 ± 2.37
Fixed Window 51	0.972	0.94 ± 0.79	0.14 ± 1.23

## Conclusion

Binarization of OCT images of the choroid can provide valuable information regarding the anatomical structure of this tissue. To date, most of the applied methods rely on traditional image processing local binarization techniques. In this study, we show that the performance of these traditional binarization techniques relies on the appropriate selection of window size, in order to achieve optimal separation of luminal and interstitial regions of the choroid, which also has an impact on the subsequent extraction of the CVI. Although not considered in previous work, manual window size selection by an experienced examiner can improve performance, and our results indicate this is a highly repeatable process (ICC = 0.968) for selection of window sizes between repeat scans. This is comparable with the interobserver repeatability reported (ICC = 0.942) in a recent study, that used the same window selection tool.^41^ However, this manual process is time-consuming, so the application of automatic techniques for image binarization is of interest. Therefore, in this study, we explored the use of a range of deep learning methods for this image analysis task.

From all tested methods, the U-Net and SegNet networks showed the highest total accuracy, with values above 96%. This indicates that more than 96% of the choroidal pixels were properly labeled as interstitial or luminal. The fixed window size showed a poorer total accuracy of 94%, with worse performance detecting interstitial tissue. Overall, all methods displayed comparable repeatability relative to the ground truth. For the CVI, SegNet showed the highest level of agreement (smallest difference) with the ground truth, and a high level of repeatability (smallest mean absolute difference between repeated scans). Repeatability data were similar across all networks. Despite the positive performance of the method, it is worth noting that the data set included OCT images from children without ocular disease, so further testing in older populations or in the presence of ocular disease may further validate the proposed methods. Similarly, given the excellent performance on the method using well-established deep learning architectures, it is likely that application of newer variations and fine tuning of network parameters (such as filter^42^ and encoder depth^43^) could further improve performance.

The potential of deep learning methods for a range of image analysis tasks have been demonstrated previously, with state-of-the-art performance for choroidal segmentation.^20^^–^^22^ The use of this method for the binarization of the choroid has not been explored comprehensively. In this work, a detailed exploration of deep learning methods for this application was undertaken and the results suggest that well-established networks can be applied to this task with excellent performance (both accuracy and repeatability). These findings provide a stepping-stone for the development of deep learning tools to binarize the choroid in OCT images for various clinical and research applications.

## Supplementary Material

Supplement 1
