# Supplementary material for: Application of Deep Learning Methods for Binarization of the Choroid in Optical Coherence Tomography Images
Source: Transl Vis Sci Technol. 2022 Feb 14;11(2):23. doi: 10.1167/tvst.11.2.23 (PMC8857621; doi:10.1167/tvst.11.2.23)
Supplement: Supplement 1 [file tvst-11-2-23_s001.pdf]

Supplementary materials

Supplementary Table 1. Testing results for un-masked images.

<table>
<tr><th colspan="2" rowspan="2"></th><th rowspan="2">Total accuracy (%)</th><th rowspan="2">IoU (%)</th><th colspan="3">Per-class accuracy (%)</th></tr>
<tr><th>Lumen</th><th>Interstitial</th><th>Sclera</th></tr>
<tr><td colspan="2">U-Net</td><td>92.47 ± 3.65</td><td>86.28 ± 8.39</td><td>94.07 ± 5.38</td><td>84.42 ± 5.91</td><td>98.94 ± 1.10</td></tr>
<tr><td rowspan="3">DeepLabV3</td><td>ResNet18</td><td>89.91 ± 2.76</td><td>81.53 ± 6.57</td><td>90.45 ± 3.98</td><td>81.48 ± 4.72</td><td>97.79 ± 2.15</td></tr>
<tr><td>ResNet50</td><td>89.97 ± 2.88</td><td>81.72 ± 6.82</td><td>90.85 ± 4.25</td><td>81.03 ± 4.90</td><td>98.04 ± 1.85</td></tr>
<tr><td>MobileNetV2</td><td>92.47 ± 3.65</td><td>86.28 ± 8.39</td><td>91.06 ± 4.51</td><td>80.35 ± 5.15</td><td>97.72 ± 1.72</td></tr>
<tr><td colspan="2">SegNet</td><td>91.93 ± 2.76</td><td>84.22 ± 6.67</td><td>92.78 ± 4.37</td><td>86.51 ± 4.48</td><td>96.52 ± 2.51</td></tr>
</table>

Supplementary Table 2. Repeatability testing for un-masked images.

<table>
<tr><th colspan="2"></th><th>Lumen (%)</th><th>Interstitial (%)</th><th>Sclera (%)</th></tr>
<tr><td colspan="2">U-Net</td><td>0.59 ± 0.55</td><td>0.57 ± 0.67</td><td>0.75 ± 0.69</td></tr>
<tr><td rowspan="3">DeepLabV3</td><td>ResNet18</td><td>0.75 ± 0.80</td><td>0.57 ± 0.57</td><td>0.78 ± 0.85</td></tr>
<tr><td>ResNet50</td><td>0.75 ± 0. 84</td><td>0.61 ± 0.56</td><td>0.90 ± 0.98</td></tr>
<tr><td>MobileNetV2</td><td>0.70 ± 0.72</td><td>0.79 ± 0.75</td><td>0.81 ± 0.95</td></tr>
<tr><td colspan="2">SegNet</td><td>0.76 ± 0.80</td><td>0.66 ± 0.65</td><td>1.03 ± 0.98</td></tr>
</table>

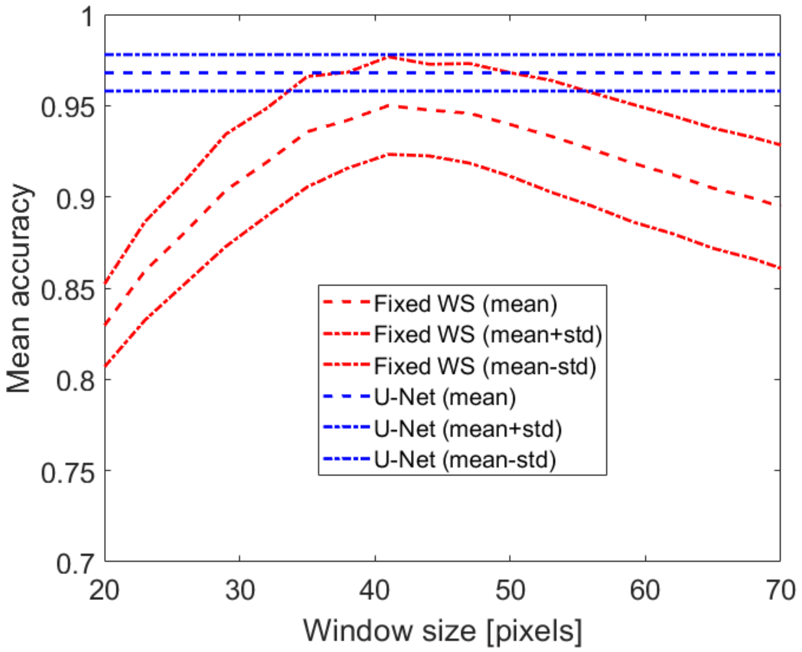

Supplementary Figure 1. Comparison of mean accuracy of a fixed window size approach (red) versus the U-Net (blue) performance. The values are reported as mean +/- one standard deviation.
